# OA31 Vitamin C deficiency presenting as juvenile idiopathic arthritis in an autistic child: a case study

**DOI:** 10.1093/rap/rkae117.031

**Published:** 2024-11-05

**Authors:** Sobia Siraj

**Affiliations:** Evelina London Children’s Hospital, Guy’s and St Thomas’ NHS Trust, London, United Kingdom

## Abstract

**Introduction:**

Considered rare in the UK, we present a case of scurvy presenting as juvenile idiopathic arthritis (JIA). Scurvy is caused by vitamin C deficiency; the incidence is reported as 0.48 cases per 100,000 children under 16 years of age (2015–2017) in the UK. Scurvy is associated with gingival hypertrophy, petechiae, and musculoskeletal pain. The musculoskeletal manifestations and systemic features can present as JIA. This case highlights the importance of considering nutritional deficiencies in the differential diagnosis of paediatric patients presenting with symptoms similar to JIA to avoid misdiagnosis and ensure appropriate management.

**Case description:**

A seven-year-old child with autism presented to the A/E with a week’s history of limping. Hip X-rays and blood tests were normal, so he was discharged. A week later, he returned with worsening symptoms, including swelling of the right knee. Blood tests and ultrasounds of bilateral hips and knees were normal, and he was once again discharged.

On Day 14 of the illness, he was readmitted with increased right knee swelling and fever. Blood tests showed elevated inflammatory markers (ESR 26 mm/hr, CRP 33 mg/L). He was commenced on IV ceftriaxone for suspected osteomyelitis, but continued to experience low-grade fevers and developed swelling in the opposite knee, right ankle and mid-foot.

On Day 22 of his illness, he was transferred to the Evelina and was found to have polyarthritis, reduced mobility, gingival hypertrophy and fever. Bloods revealed dropping counts, Hb 74 g/L, WBC 1.9 x 10^9/L and neutrophils 0.5 x 10^9/L, along with rising ferritin (96 -> 301 µg/L), dropping ESR (63 -> 17 mm/hr), and elevated LDH (428 U/L). Immunophenotyping and blood films were negative, leading to a provisional diagnosis of juvenile idiopathic arthritis (JIA) with evolving macrophage activation syndrome (MAS). He was started on anakinra and IV methylprednisolone.

A full-body MRI revealed extensive metaphyseal signal abnormalities and mild soft-tissue oedema without active synovitis or joint effusion. The differentials included CRMO, lymphoproliferative disease, and scurvy. By this time, the lab Vitamin C level returned as extremely low at < 2.8 (ref 26-84 µmol/L).

Following vitamin C supplementation and dietary support, his full blood count, ferritin, ESR and CRP normalised. He was gradually weaned off anakinra and steroids. His joint swelling, fever, and pain resolved, and he began to remobilise. The patient was discharged under the care of community paediatrics, a dietitian, and the physiotherapy team.

**Discussion:**

Scurvy results from a severe deficiency of vitamin C in the diet. It is less common than other nutritional deficiencies, often leading to delayed diagnosis, particularly in developed countries. Children with unusual eating habits, global developmental delay or social communication disorders are at higher risk of developing this condition. This was an intriguing case of polyarthritis associated with scurvy.

Vitamin C is essential for the synthesis of collagen, carnitine and certain neurotransmitters. It plays a critical role in immune function and the absorption of iron from the gut. Prolonged dietary insufficiency of vitamin C can lead to impaired collagen synthesis. In approximately 80% of instances, scurvy presents with musculoskeletal symptoms such as joint pain, muscle pain, haemarthrosis, and muscle haematomas.

Gingival hypertrophy in a child with significant autism was the trigger to investigate vitamin C levels in our patient with polyarthritis. The eventual diagnosis of scurvy was confirmed by low vitamin C levels combined with the MRI findings. This case underscores the importance of considering nutritional deficiencies in patients with complex presentations and highlights the need for a thorough dietary history.

**Key learning points:**

• **Importance of dietary history:** The primary learning point from this case is the critical importance of obtaining a comprehensive dietary history in paediatric patients, particularly those with atypical eating habits or developmental delay/social communication disorders.

• **Importance of a thorough clinical examination:** The gingival hypertrophy in the case was the giveaway to check vitamin C levels.

• **Differential diagnosis of musculoskeletal symptoms:** This case highlights the necessity of including nutritional deficiencies, such as scurvy, in the differential diagnosis of musculoskeletal symptoms like joint pain and swelling.

• **Role of a multidisciplinary approach:** The successful management of this case underscores the importance of a multidisciplinary approach. Involving specialists from rheumatology, orthopaedics, nutrition, and general paediatrics was essential in reaching the correct diagnosis and providing comprehensive care. This collaborative effort ensured that all aspects of the patient’s condition were addressed.

• **Early intervention and treatment:** Early intervention with vitamin C supplementation led to a rapid resolution of symptoms and normalisation of inflammatory markers. This case demonstrates the effectiveness of timely treatment in preventing long-term complications and improving patient outcomes. It also highlights the importance of considering vitamin supplementation in patients at risk of dietary deficiencies. The WHO recommends all children under 5 are supplemented with multivitamins.

